# Perceptions and Experiences of Undergraduate Medical Students Regarding Social Accountability: a Cross-sectional Study at a Subsaharan African Medical School

**DOI:** 10.21203/rs.3.rs-3756902/v1

**Published:** 2023-12-19

**Authors:** Lorraine Oriokot, Ian Guyton Munabi, Sarah Kiguli, Aloysius Gonzaga Mubuuke

**Affiliations:** Makerere University

**Keywords:** Social accountability, medical school, medical education, medical students, community-based education research and service

## Abstract

**Background:**

Medical schools are called to be socially accountable as a feature of excellent medical education. Medical students are essential to the development of socially accountable medical schools. Therefore, understanding the perceptions and experiences of medical students regarding social accountability is critical for efforts to improve social accountability practices and outcomes.

**Methods:**

This cross-sectional online questionnaire-based survey used Google Forms and involved medical students in their fourth and fifth years of study at the Makerere University School of Medicine. The survey was conducted between September 2022 and October 2023. We used a study questionnaire and the Students’ toolkit for social accountability in medical schools to collect data on demographics, perceptions and experiences and evaluate social accountability.

**Results:**

A total of 426 medical students responded to the online questionnaire. The mean age of the students was 25.24 ± 4.4 years. Most of the students were male (71.3%), and most were in their fourth year of study (65%). Most of the students (43.66%) evaluated the school as having a good level of social accountability. The evaluation items referring to community-based research and positive impact on the community had the highest mean scores. Only 6 (3.64%) students provided an accurate definition of social accountability. Students receiving career guidance in secondary school was associated with evaluating social accountability in the medical school as strong (p-0.003).

**Conclusions:**

Medical students evaluated the medical school favorably in terms of social accountability. Receiving career guidance in secondary school was significantly associated with a positive evaluation of social accountability.

## Background

Medical students are essential to the development of a socially accountable medical school (1, 2). Social accountability for medical schools is defined as the obligation of the medical school to direct education, research, and service activities toward the most important needs of the community served by the medical school and its graduates (3). While medical students are expected to learn about social accountability and become socially accountable practitioners, little has been documented about their perceptions and experiences of social accountability.

Medical students have distinct and unique experiences during training, which may influence their future practice and choices following graduation (4, 5). It is thus important to understand these experiences to improve the teaching and learning of social accountability. Previous studies have shown that medical students have a limited understanding of social accountability (5–7). A survey of Deans in Korea reported that interactions between partners had the greatest influence on social accountability in medical education (8). Medical students are thus key partners for social accountability; therefore, understanding their perceptions and experiences could be a starting point for improving their learning and adoption of social accountability (1).

The Students’ Toolkit on Social Accountability of Medical Schools was developed through a collaboration between the International Federation of Medical Students Associations (IFMSA) and the Training for Health Equity Network (THEnet) (4). The toolkit aims to provide a brief introduction to social accountability for medical students and empower them to make a difference in their schools in the area of social accountability. The toolkit is an evaluation tool for students to assess the progress made by their medical school in terms of social accountability and to create action plans to improve social accountability at the medical school (1). This kit has been applied in various settings, especially in high-income countries (9, 10).

The perceptions and experiences of medical students regarding social accountability are unique because of contextual differences that may influence their learning and adoption of social accountability (11, 12). There is a dearth of published literature about these perceptions and experiences, particularly from sub-Saharan Africa. The purpose of this study therefore was to determine the perceptions and experiences of medical students regarding social accountability at the Makerere University School of Medicine.

## Methods

### Study design and questionnaire:

A cross-sectional online questionnaire-based survey was conducted between September 2022 and October 2023 using Google Forms. This study collected responses from medical students from the Makerere University School of Medicine. The validated Students’ Toolkit on Social Accountability in Medical Schools, which has been applied widely to study perceptions of social accountability, was used for this study, with some modifications. The final study questionnaire had three sections: demographic information, perceptions, and experiences related to social accountability; and the evaluation section from the toolkit. The questionnaire was pretested on eight undergraduate medical students in their fourth or fifth year of study. The link to the questionnaire on Google Forms was sent to each student’s email. Each link was unique to each email address to avoid reuse and double enrollment.

### Study setting

The study was conducted at the Makerere University Medical School in Uganda. Makerere University is a government-owned university, and the Makerere University School of Medicine is the oldest medical school in East Africa. The Bachelor of Medicine and Bachelor of Surgery (MBCHB) program spans five years. Medical student recruitment is performed at the national level and is based mainly on academic performance (13). Following graduation, students undertake one year of supervised internship. After successful completion of the internship period, graduates can obtain full registration status with the Uganda Medical and Dental Practitioners Council and thereafter practice as medical doctors. At this point, the doctor may decide to continue as a general practitioner or take on further postgraduate training in a field of choice, such as Family Medicine, Obstetrics and Gynecology, Internal Medicine, General Surgery or other fields.

### Characteristics of participants

All students in their fourth or fifth year of medical school were invited to participate in the study through trained research assistants. Fourth- and fifth-year students were selected because they were more likely to have had experiences related to social accountability in medical school. The research assistants were fourth- and fifth-year students who were trained in the study procedures. The research assistants obtained written informed consent from the participants and registered their email addresses. The links to the electronic survey were sent to the registered email addresses. Students who did not initially respond to the survey were reminded by the research assistant. The link was made available in two academic years, allowing two sets of fourth-year students to respond and one set of fifth-year students since these had been enrolled in the previous year.

### Statistical analysis

The data collected in Google Forms were exported to Microsoft Excel, checked for completeness and missing data, and then cleaned. The data were analyzed with R statistical software. The descriptive statistics are reported. The total score for the students’ toolkit 12 social accountability evaluation items was computed, and the means and standard deviations for the individual items are presented. The total scores were categorized according to the key provided in the student toolkit. The categories included 0–8, weak foundation; 9–17, some evidence of social accountability; 18–26, identify areas of improvement; and 27–36, strong foundation. The categories were divided into limited social accountability (weak and some social accountability) and strong social accountability (looking for areas of improvement and strong social accountability), and regression analysis was conducted to determine the associated factors. The chi-square test and Fisher’s exact test were used. A p value < 0.05 indicated statistical significance.

## Results

This study involved 426 medical students in their fourth or fifth year of study at the Makerere University School of Medicine. The mean age of the students was 25.24 ± 4.4 years. Most of the participants were in their fourth year of study, as the link was available over two academic years. The characteristics of the study participants are summarized in Table 1.

### Student perceptions and experiences of social accountability

**Figure 1:**
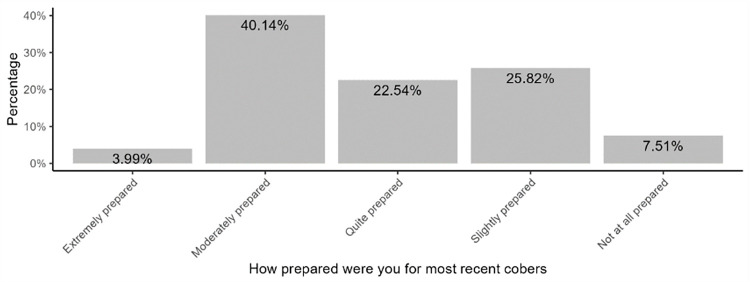
Level of Preparedness for Last Community-Based Education Research and Service Experience

When asked if they had ever heard about social accountability, 165 (38.73%) students responded ‘yes’. However, only 6 (3.64%) of those who reported having heard about social accountability provided an accurate definition of the term. Of the 165 (39%) participants who reported hearing about social accountability before, 91 (55%) encountered the term in personal reading. The average time spent in community-based education research and service was 6.8 weeks, and most (40.14%) of the students reported feeling moderately prepared for their last COBERS experience. (Fig. 1)

### Students’ evaluation of social accountability at medical schools

Using the Students’ toolkit for social accountability in medical schools, 48.12% of the medical students evaluated the medical school as having good social accountability, with a total score between 18 and 26. In contrast, 1.41% of the students felt that the medical school had a weak foundation for social accountability.

Of the twelve items used to assess social accountability, seven items (1, 2, 3, 7, 8, 11 and 12) were related to perceptions, while five items (4, 5, 6, 9 and 10) were related to experiences. Item 10 (Does your school have community-based research?) 2.57 ± 0.62 and Item 12 (Does your school have a positive impact on the community?) 2.434 ± 0.67 had the highest mean scores. Item 4 (Do you learn about other cultures?) 1.37 ± 0.93 and Item 8 (Do your teachers reflect the sociodemographic characteristics of the reference population?) 1.60 ± 0.92 had the lowest mean scores. (Table 2)

The only factor that was significantly associated with evaluation of social accountability in medical school as strong was receiving career guidance in secondary school (p 0.003). (Table 3)

## Discussion

We evaluated the perceptions and experiences of medical students at the Makerere University School of Medicine regarding social accountability. Our study findings suggest that medical students at the Makerere University School of Medicine have varied perceptions and experiences of social accountability; most of the students evaluated the medical school favorably, and receiving career guidance in secondary school was associated with a positive evaluation of social accountability. Most medical students felt that the medical school had a good level of social accountability and needed to look at areas of weakness and ways to advocate for improving social accountability. The highest mean scores for items in the students’ toolkit for social accountability in medical schools were for community-based research and for the positive impact of the medical school on the community. The least favorable assessment items include learning about other cultures and teachers reflecting the reference population. Medical students come from varied backgrounds and have different experiences during medical education; therefore, it is not surprising that the perceptions, experiences and evaluations of the medical school were diverse. Career guidance may have helped the students choose an appropriate career path, leading to more positive perceptions and experiences for students who had received career guidance prior to medical school. This study helps us to better understand medical students’ views regarding social accountability and the factors that may influence these views. These findings provide a starting point for improving student experiences of social accountability in medical education.

The high percentage of students who gave a good evaluation of social accountability at medical school reflects efforts by the medical school to achieve this goal. These efforts include community-based education, research and services and adopting a competency-based medical education curriculum to better meet the community’s needs (14, 15). Our findings are comparable to those of a study conducted at a Saudi Arabian government-funded medical school where most students felt that the medical school was performing well in terms of social accountability (4).

There have been gains in pursuing social accountability goals, and more needs to be done to enable students to understand the concept and demonstrate its values. Our findings concur with a previous study that showed a poor understanding of social accountability among stakeholders. This previous study also provided evidence of social accountability in medical school activities (6). Similarly, a qualitative study in the United Kingdom also showed that students did not understand the concept of social accountability or feel that it has implications for their medical education or future practice (5).

Medical students are central to social accountability efforts in medical schools. The perceptions and experiences of the medical students in this study reflect exposure to the concept and practice of social accountability. Learning social accountability requires deliberate and meaningful efforts in which medical students are considered partners.

The major strength of this study lies in the relatively large sample size, as we tried to enroll all eligible participants. The limitation of this study is that it was conducted at one study site, which may limit the generalizability of the findings. However, the presence of similar findings in other settings supports the generalizability of our findings. Recall bias was minimized by enrolling students who were still in medical school.

## Conclusions

Medical students at the Makerere University School of Medicine have varied perceptions and experiences of social accountability. Most students felt moderately prepared for COBERS and evaluated medical school positively for social accountability. Regarding individual items in the student toolkit, the presence of community-based research and the school having a positive impact on the community had higher mean evaluation scores. Receiving career guidance in secondary school was associated with a positive evaluation of the medical school.

### Recommendations

The medical school should provide students with more opportunities to learn about social accountability and routinely evaluate the perceptions and experiences of medical students regarding social accountability. Students should be better prepared for Community-Based Education Research and Service.

## Figures and Tables

**Figure 2:**
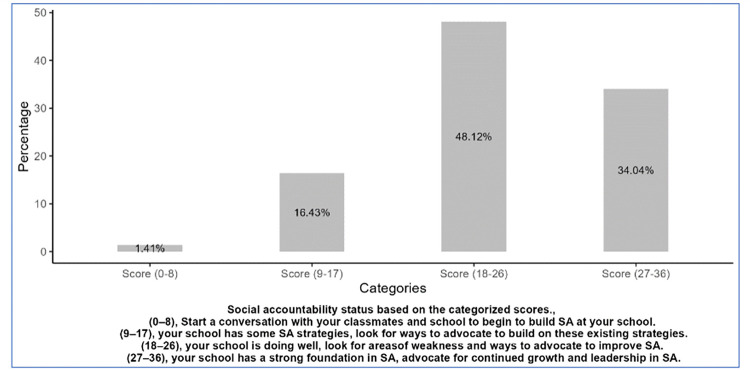
Evaluation categories of social accountability at the medical school

**Table 1:** Characteristics of the study participants

Variable	Characteristic	N = 426 (%)	(95 CI)1–2
Gender	Female	122 (28.64)	(24.44–33.23)
	Male	304 (71.36)	(66.77–75.56)
Age	20-24	283 (66.43)	(61.70–70.86)
	25-30	86 (20.19)	(16.54–24.38)
	31-35	32 (7.51)	5.272–10.55)
	36-47	25 (5.87)	(3.341–7.833)
Tribe	Muganda	158 (37.26)	3.910–8.656)
	Munyankole	51 (12.03)	(9.164–15.60)
	Other tribe	215 (50.71)	(45.85–55.56)
Nationality	Ugandan	410 (96.24)	(93.84–97.77)
	Other Nationality	16 (3.76)	(2.235–6.156)
Highest education level before medical school	A level and equivalent	317 (74.41)	(69.94–78.44)
	Bachelor	36 (8.45)	(6.066–11.61)
	Diploma	73 (17.14)	(13.75–21.13)
Year of study	Year 4	276 (64.79)	(60.02–69.29)
	Year 5	150 (35.21)	(30.71–39.98)
Received career guidance in secondary school	Yes	343 (80.71)	(76.56–84.28)
	No	82 (19.29)	(15.72, 23.44)

**Table 2:** Students' evaluation of social accountability at the Makerere University School of Medicine

Question	Excellent	Good	Somewhat	No	Mean ± SD
Does your institution have a clear social mission statement around the communities that they serve?	91 (21.36)	186 (43.66)	109 (25.59)	40 (9.39)	1.77 ± 0.891
Does your curriculum reflect the needs of the population you serve?	99 (23.24)	216 (50.70)	89 (20.89)	22 (5.16)	1.92 ± 0.802
Does your school have community partners or stakeholders who shape your school?	87 (20.42)	164 (38.50)	120 (28.17)	55 (12.91)	1.664 ± 0.944
Do you learn about other cultures and other social circumstances in medical context in your curriculum?	53 (12.44)	133 (31.22)	157 (36.85)	83 (19.48)	1.366 ± 0.934
Do the places/locations you learn at in practice include the presence of the populations that you will serve?	161 (37.79)	174 (40.85)	74 (17.37)	17 (3.99)	2.124 ± 0.835
Are you required to do community-based learning (opposed to only elective opportunities)?	211 (49.53)	146 (34.27)	44 (10.33)	25 (5.87)	2.275 ± 0.872
Does your class reflect the sociodemographic characteristics of your reference population?	101 (23.71)	175 (41.08)	99 (23.24)	51 (11.97)	1.765 ± 0.946
Do your teachers reflect the sociodemographic characteristics of your reference population?	71 (16.67)	170 (39.91)	129 (30.28)	56 (13.15)	1.601 ± 0.915
Does your learning experience also provide an active service to your community?	145 (34.04)	193 (45.31)	76 (17.84)	12 (2.82)	2.106 ± 0.789
Does your school have community-based research?	270 (63.38)	132 (30.99)	21 (4.93)	3 (0.70)	2.57 ± 0.622
Does your school encourage you to undertake generalist specialties (eg. family medicine, general practice)?	153 (35.92)	123 (28.87)	94 (22.07)	56 (13.15)	1.876 ± 1.045
Does your school have a positive impact on the community?	225 (52.82)	164 (38.50)	34 (7.98)	3 (0.70)	2.434 ± 0.67

**Table 3:** Factors associated with students' evaluation of social accountability

Variable	Characteristic	Overall, N = 426	Limited SA, N = 76	Strong SA, N = 350	p value
Gender	Female	122	28 (22.95)	94 (77.05)	0.081
	Male	304	48 (15.79)	256 (84.21)	
Receiving career guidance in secondary school	Yes	344	52 (15.12)	292 (84.88)	0.003
	No	82	24 (29.27)	58 (70.73)	
Year of study	Year 4	276	47 (17.03)	229 (82.97)	0.6
	Year 5	150	29 (19.33)	121 (80.67)	
Age category	19–24	283	52 (18.37)	231 (81.63)	0.7
	25–47	143	24 (16.78)	119 (83.22)	
Nationality	Ugandan	410	75 (18.29)	335 (81.71)	0.3
	Other	16	1 (6.25)	15 (93.75)	
Education level prior to medical school	A-level and equivalent	317	57 (17.98)	260 (82.02)	0.9
	Graduate	109	19 (17.43)	90 (82.57)	
CGPA category	2.36–3.99	352	61 (17.33)	291 (82.67)	0.5
	4.00–5.00	67	14 (20.90)	53 (79.10)	

## Data Availability

The datasets used and/or analyzed during the current study are available from the corresponding author upon reasonable request.

## Abbreviations

COBERS: Community-Based Education Research and Service
